# Erratum: ACF7 regulates inflammatory colitis and intestinal wound response by orchestrating tight junction dynamics

**DOI:** 10.1038/ncomms16121

**Published:** 2017-07-11

**Authors:** Yanlei Ma, Jiping Yue, Yao Zhang, Chenzhang Shi, Matt Odenwald, Wenguang G. Liang, Qing Wei, Ajay Goel, Xuewen Gou, Jamie Zhang, Shao-Yu Chen, Wei-Jen Tang, Jerrold R. Turner, Feng Yang, Hong Liang, Huanlong Qin, Xiaoyang Wu

Nature Communications 8: Article number: 15375; DOI: 10.1038/ncomms15375 (2017); Published: 05 25 2017; Updated: 07 11 2017

The affiliation details for Yanlei Ma and Yao Zhang are incorrect in this Article. The correct affiliation details for these authors are given below:

Yanlei Ma: 

 Department of GI surgery, Shanghai Tenth People's Hospital Affiliated with Tongji University, 301 Yanchang Road, Shanghai 200072, China. 

 The University of Chicago, Ben May Department for Cancer Research, Chicago, Illinois 60637, USA. 

 Department of Colorectal Surgery, Fudan University Shanghai Cancer Center, Shanghai, China.

Yao Zhang: 

State Key Laboratory Cultivation Base for the Chemistry and Molecular Engineering of Medicinal Resources, Ministry of Science and Technology of China, Guanxi Normal University, Guilin 541004, China.

