# Supplementary material for: ACF7 regulates inflammatory colitis and intestinal wound response by orchestrating tight junction dynamics
Source: Nat Commun. 2017 May 25;8:15375. doi: 10.1038/ncomms15375 (PMC5458510; doi:10.1038/ncomms15375)
Supplement: Supplementary Information — Supplementary Figures and Supplementary Table [file ncomms15375-s1.pdf]

Supplementary Figure 1

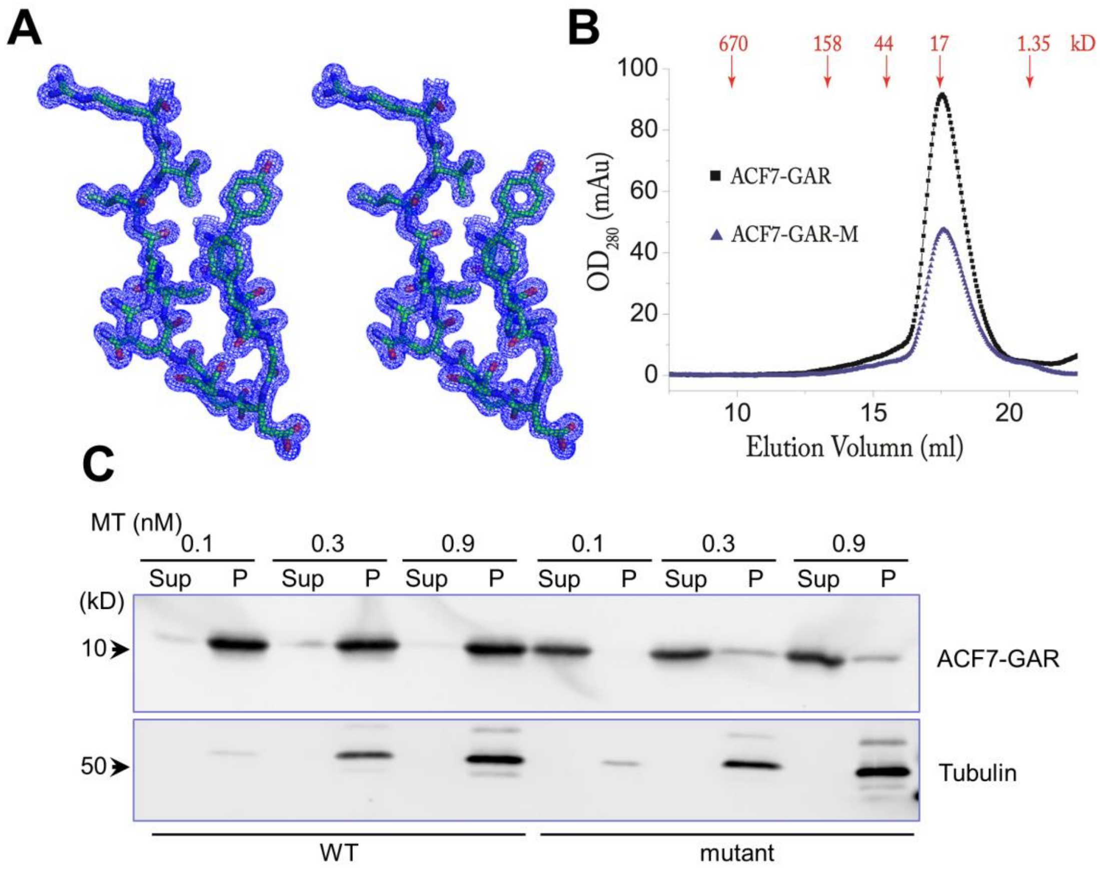

Supplementary Figure 1: ACF7 associates with microtubules through its GAR domain. (A) The stereo image of a portion of the electron density map of ACF7-GAR contoured at 1.2 σ. **(B)** Profile of superdex 200 10/300 GL chromatography of purified GAR and GAR mutant of ACF7. The data shows that the proteins are monodispersed. The peak of GAR (calculated molecular weight, 9.4 kD) elutes at ~ 17 kD, suggesting that it behaves as a dimer. **(C)** Interaction between GAR domain and microtubules was determined by co-precipitation assay with varying amount of microtubules. Supernatants (Sup) and pellets (P) after ultracentrifugation were subjected to immunoblots with different antibodies as indicated.

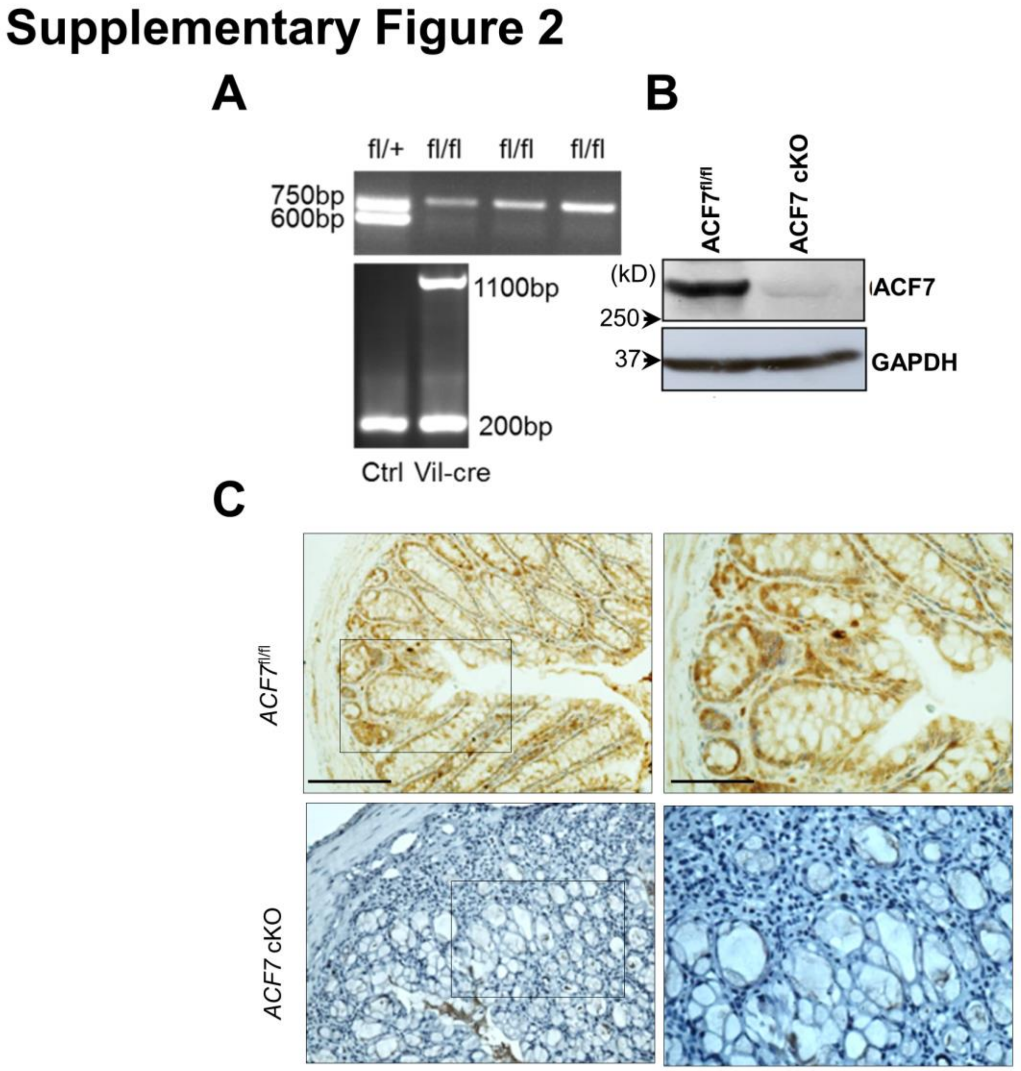

Supplementary Figure 2: Generation of *ACF7* cKO mouse model. (A) Representative PCR genotyping results for *ACF7* cKO with vil-Cre. **(B)** Western blotting analysis indicates loss of *ACF7* expression in colon epithelium in cKO animals. **(C)** Immunohistochemistry indicates loss of ACF7 staining in intestinal epithelium in the cKO animals. Boxed areas are enlarged and shown at the right side. Scale bar represents 200 µm (left panel) or 50 µm (right panel).

Supplementary Figure 3

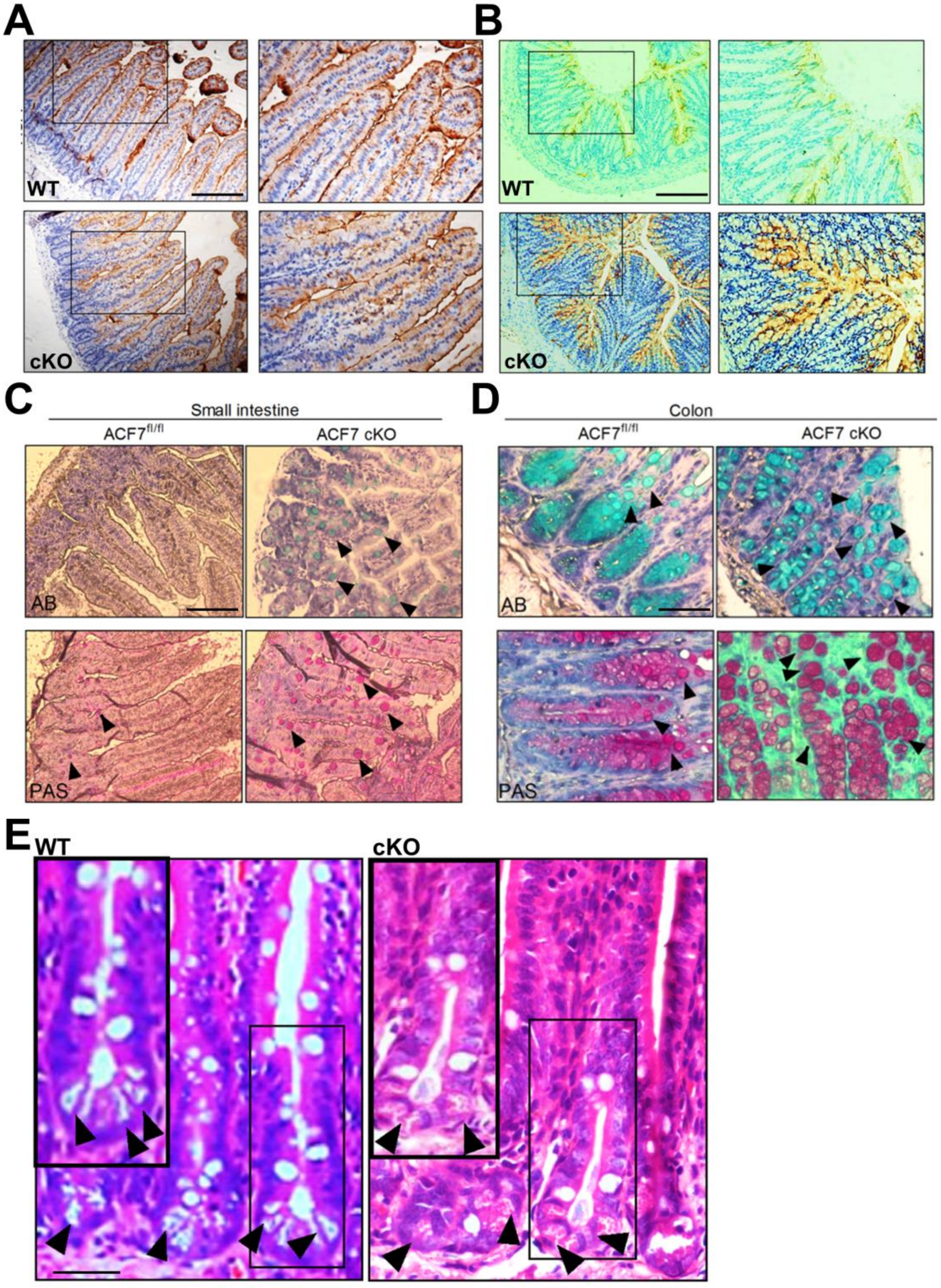

Supplementary Figure 3: Differentiation of intestinal epithelial cells in *ACF7* cKO animals. (A-B)

Immunohistochemical staining with the small intestinal differentiation marker (intestinal alkaline

phosphatase, ALP) (A) and colonic differentiation marker (carbonic anhydrase-I, CA-1) (B). Note that there was no significant difference in enterocyte and colonocyte differentiation between the cKO animals and WT animals (magnification, ×200 and ×400). Scale bar represents 50 μm. **(C-D)** Paraffin-embedded colon sections were stained with various epithelial markers to examine intestinal differentiation. Alcian blue (AB) and Periodic acid-Schiff (PAS) staining identified goblet cells (black arrows) in the small intestine (A) and colon (B). Scale bar represents 50 μm. **(E)** HE staining showed Paneth cells (indicated by the black arrow) in the crypts of WT and *ACF7* cKO mice (magnification, ×400). Scale bar represents 20 μm.

Supplementary Figure 4

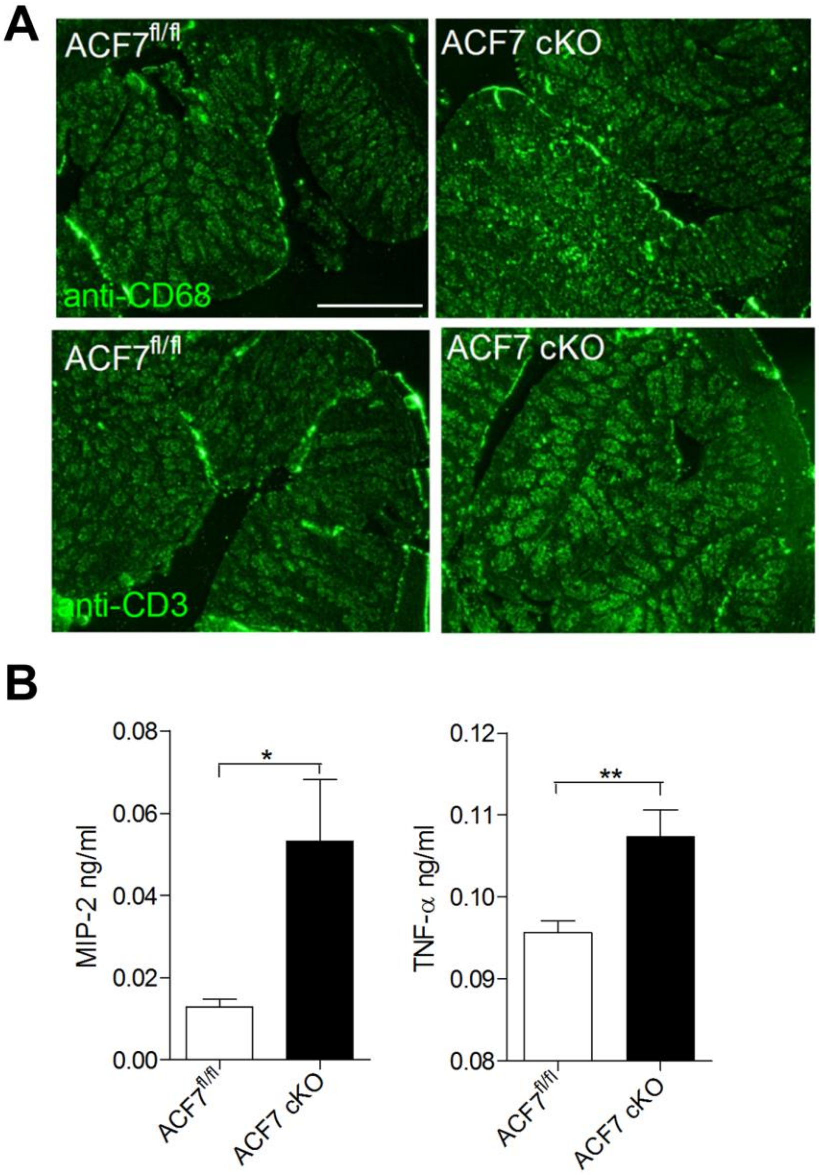

Supplementary Figure 4: Inflammatory infiltration upon DSS treatment. (A) Immunofluorescence staining demonstrates infiltration of $CD68^+$ and $CD3^+$ immune cell infiltration in WT and *ACF7* cKO colon after DSS treatment. Scale bar represents 200 µm. **(B)** The expression levels of MIP-2 and TNF-α in serum of *ACF7* cKO mice and WT controls after DSS treatment (means ± SEM, n=6, *: P<0.01, **: P<0.05, student t-test).

Supplementary Figure 5

A

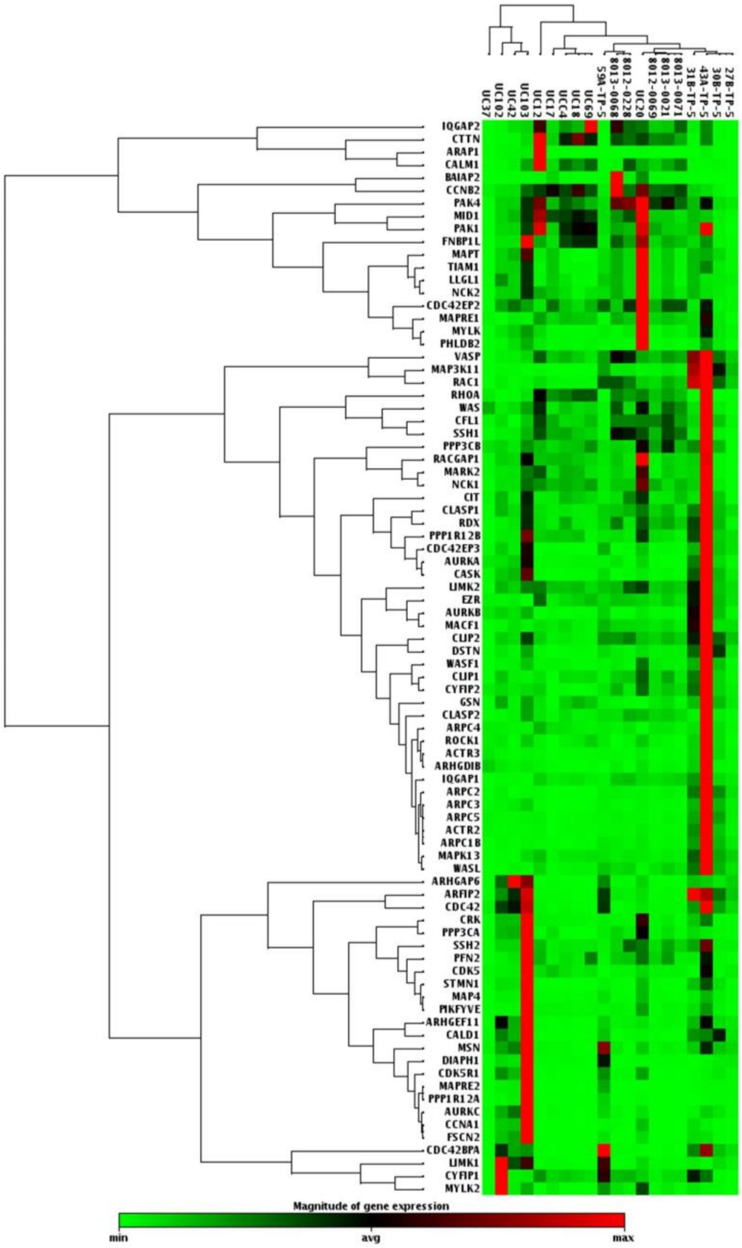

B

Gene Name	Fold Change
ARPC1B	**0.25**
ARPC2	**0.13**
CDK5	**2.35**
CLIP2	**0.22**
DSTN	0.25
IQGAP1	**0.31**
MACF1/ACF7	**0.34**
MAP3K11	**0.04**
MID1	**2.85**
RAC1	**0.01**
VASP	**0.24**
WASL	**0.12**
ACTB	**0.29**
B2M	**0.21**
RPLP0	**0.15**

Supplementary Figure 5: Clustering analysis for human cytoskeletal regulator PCR array. **(A)** Clustering analysis of PCR array for human cytoskeletal regulators in healthy and UC patients. **(B)** List of candidate genes that display fold changes over 2.

Supplementary Figure 6

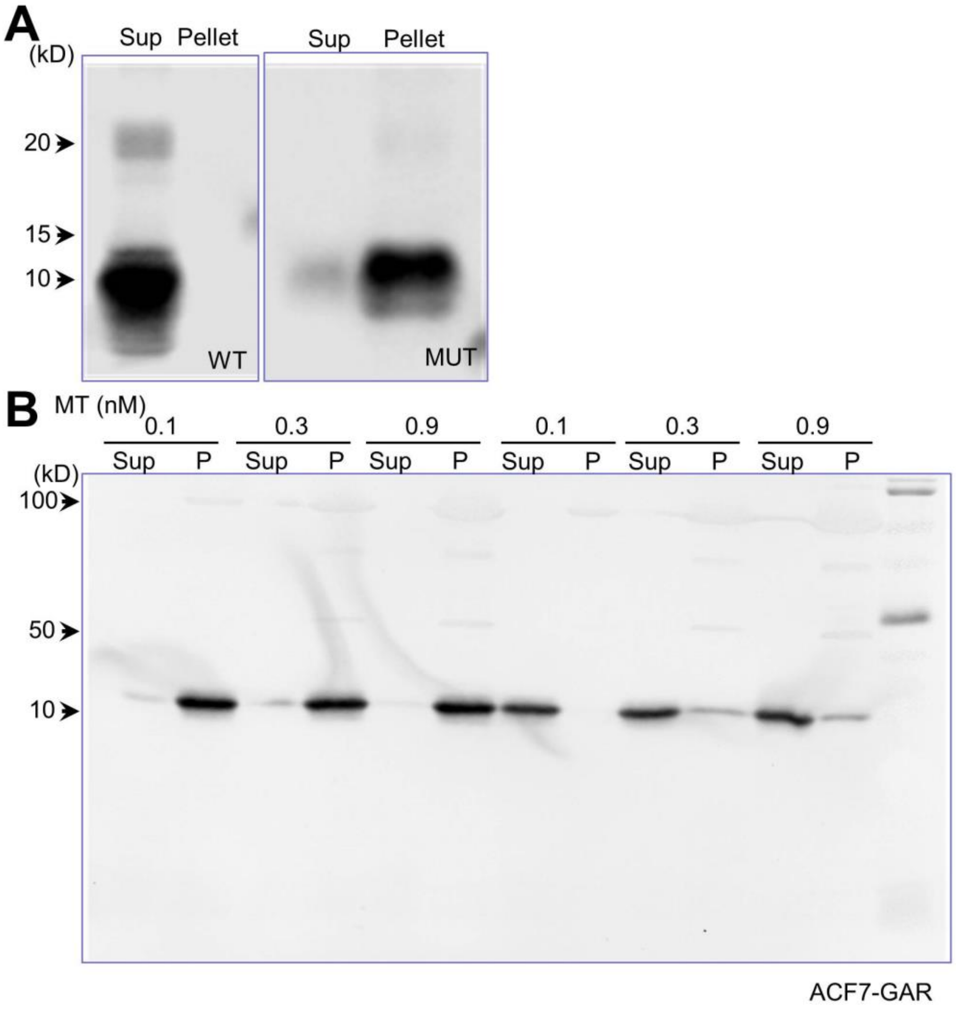

Supplementary Figure 6: Uncropped scan of immunoblots. **(A)** Uncropped scan of immunoblotting results in Fig. 2F (WT and mutant protein precipitated by microtubules). **(B)** Uncropped scan of immunoblots in Supplementary Fig. 1B (top panel).

Supplementary Table 1 Associations between demographics and the expression level of MACF1 in the patients with UC

	Low MACF1	High MACF1	P-value
	n = 20	n = 22	
**Age (year)**			0.217
< 50	14	7	
≥ 50	6	15	
**Age (year), mean ± SD**	44.10 ± 20.81	49.73 ± 16.68	0.337
**Sex**			0.380
Female	9	7	
Male	11	15	
**Extent of disease of resected colon**			0.592
Proctitis or left-sided	14	18	
Total	6	4	
**Geboes grades**			0.027
2	1	3	
3	0	3	
4	1	3	
5	18	13	
**ESR (mm/h), mean ± SD**	35.82 ± 16.87	25.14 ± 15.66	0.198
**CRP (mg/L), mean ± SD**	42.12 ± 48.89	13.48 ± 13.90	0.046
**Leukocyte (WBC, ×10^9) , mean ± SD**	6.79 ± 2.35	7.70 ± 2.97	0.317
**Hemoglobin (g/L), mean ± SD**	114.24 ± 26.73	119.29 ± 20.92	0.543
**Albumin (g/L), mean ± SD**	35.19 ± 5.84	38.67 ± 4.03	0.065
**FOBT**			0.025
Negative	11	19	
Positive	9	3	

ESR, erythrocyte sedimentation rate; CRP, C reactive protein; FOBT, fecal occult-blood test; The median value of the expression levels of MACF1 was set as a cutoff point for categorized the UC patients.

The immunostaining grading was performed based on the staining extent (percentage of positive cells graded on scale from 0 to 3: 0, none; 1, 1 - 30%; 2, 31 - 60%; 3, > 60%) and the staining intensity (graded on scale from 0 to 3: 0, none; 1, mild/weak; 2, moderate; 3, strong). The combination of extent (E) and intensity (I) of staining (E × I) was gained varying from 0 to 9.

Supplementary Table 1: Associations between demographics and the expression level of *ACF7* in the patients with UC.
